# Hydrate Chloral

**Published:** 1871-01

**Authors:** S. W. Gould

**Affiliations:** Argos, Ind.


Article IV. — *Hydrate Chloral.* By S. W. Gould, M.D., Argos, Ind.

As a number of physicians have been giving their experience in the use of Hydrate Chloral, I will briefly give you mine. When the article was first introduced to the profession, I was afraid to administer it in larger doses than 10 grains to 15 grains, and as a consequence no visible effects were produced. I was disposed to pronounce it a *humbug*, and return to opium and its preparations to produce sleep and relieve pain. Soon after I was attacked with a very severe headache, for which I concluded to try the effects of the Hydrate Chloral in larger doses than I had been giving my patients. I dissolved 40 grains in a little water—adding a few drops of essence of peppermint to destroy the unpleasant taste—and swallowed it. In a few minutes the pain was greatly relieved, though not entirely, and I felt as happy as a king at his coronation. In the course of an hour the pain returned with all its former severity. I weighed out 60 grains and took it. In less than five minutes I was in the land of Morpheus (or *hydra-chloralus*), and the four hours which followed are a perfect blank in my existence. When I awakened the pain was gone, and did not return, nor were there any unpleasant results.

On the evening of the 2nd inst. my wife was attacked with tic doloureux (to which she is subject), and the pain was more intense than ever before. Opium, or any of its preparations, cannot be taken by her without the most distressing results. Nausea or vomiting will continue for twenty-four or forty-eight hours after taking even the one-eighth of a grain of morphia. I have given it to her by hypodermic injection, but the result was the same, of course. In this instance I concluded to try the Hydrate Chloral, my confidence in the agent being greatly strengthened by its effects on myself. About 7 o'clock I administered to her 40 grains. In less than ten minutes she was in a profound sleep, the breathing being more easy and quiet than in natural sleep. She lay in this easy condition for about two hours, when I aroused her for the purpose of getting her to her room. The pain returned and she went to her room screaming. I repeated the dose of 40 grains, which was followed, in five or ten minutes, by sound sleep, which continued till sunrise the following morning. The pain was gone—nothing remaining to remind her of the severe attack of the evening before but the soreness which usually succeeds an attack of this kind. Since then I have used the remedy in a half dozen similar cases, and with the happiest results. I now have almost unlimited confidence in the agent, and attribute my former failures to the small quantity given. I have talked with several physicians about the preparation, and more than half of them deny that it has any hypnotic property. Upon investigation, however, I ascertained that their failure to obtain its effects depended either on a deteriorated article, or too small doses. (Many druggists know nothing about keeping it.) As some are much more easily impressed than others, the physician should begin with the minimum dose, and increase until its effects are produced.

